# Decoding the physiological response of plants to stress using deep learning for forecasting crop loss due to abiotic, biotic, and climatic variables

**DOI:** 10.1038/s41598-023-35285-3

**Published:** 2023-05-26

**Authors:** Mridul Kumar, Zeeshan Saifi, Soami Daya Krishnananda

**Affiliations:** Department of Physics and Computer Science, Dayalbagh Educational Institute, Agra, 282005 India

**Keywords:** Abiotic, Biotic, Computational biophysics, Plant development

## Abstract

This paper presents a simple method for detecting both biotic and abiotic stress in plants. Stress levels are measured based on the increase in nutrient uptake by plants as a mechanism of self-defense when under stress. A continuous electrical resistance measurement was used to estimate the rate of change of nutrients in agarose as the growth medium for *Cicer arietinum* (Chickpea) seeds. To determine the concentration of charge carriers in the growth medium, Drude’s model was used. For identifying anomalies and forecasting plant stress, two experiments were conducted and outliers were found in electrical resistance and relative changes in carrier concentration. Anomaly in the first iteration was detected by applying k-Nearest Neighbour, One Class Support Vector Machine and Local Outlier Factor in unsupervised mode on electrical resistance data. In the second iteration, the neural network-based Long Short Term Memory method was used on the relative change in the carrier concentration data. As a result of the change in resistance of growth media during stress, nutrient concentrations shifted by 35%, as previously reported. Farmers who cater to small communities around them and are most affected by local and global stress factors can use this method of forecasting.

## Introduction

Plant health can be defined as the well-being of a plant individually or community of plants and crops, along with co-existing diverse flora in natural or cultivated ecosystems^1^. Any factor that disrupts this well-being, whether biotic or abiotic, can be called stress. From the prevailing global scenario, it is apparent that climate change has emerged as one of the primary causes that can directly or indirectly affect the stress levels in plants. Future concerns raised by the policymakers on food security, availability and accessibility of nutritious food call for a timely, scalable, simple and generic method that can assist farmers in maintaining plant health and yield. This socio-agri multidisciplinary nature of the challenge works advantageously to the problem, as now food security and crop loss are just not a concern of plant pathologists, but researchers from diverse fields are coming up with multidisciplinary methods to sustain life, which is also one of the main concern in the 17 Sustainable Development Goals of the United Nations^2,3^. Prevailing extreme and unpredictable weather conditions, the COVID-19 pandemic, and the Ukraine–Russia war have clearly indicated the vulnerability of the global food market due to the dependence on giant farm holders^4^. This warrants urgently strengthening small farm holders and regional and community hubs to cater to local demands. The EAT-Lancet Commission report also suggests that shifting towards a plant-based nutritious diet is the only solution to address food insecurity^5^ particularly keeping in view the 10 billion people to be fed by 2050^6^.

As very aptly stated by Prasanna et al., a truly integrated and holistic approach is necessary to ensure sustainable integration of plant health innovations into the agri-food systems and in social landscapes, so that the technologies reach all those who are in need, especially the small farm holders^7^. It has been reported that the small farm holders having less than 2 hectares of land make up 84% of the farming community worldwide^8^. Therefore, the food security target can only be met by keeping small and marginalized farm holders as key players. Furthermore, small farm holders generally belong to the low-income group. Therefore, the technological interventions should be frugal and low cost to ensure mass adoption^9^. Crop yield can be increased either by increasing the area of farm land or by increasing the productivity on existing agricultural land (reducing the crop loss or other technological interventions). Out of these two options, increasing the crop yield is preferable as it avoids greenhouse gas emissions and large-scale disruption of existing ecosystems^10^. This requires a timely stress detection system that is frugal, and simple enough to be handled and maintained by the small farm holder themselves.

Analysis of the agricultural GDP of the World Bank suggests that technology intervention along with strategic investment in agriculture will be three times more effective in alleviating poverty in countries that highly depend on agriculture^11^ than investing in any other sector. Here it is important that small farm holders and gardeners also have access to appropriate methods of mitigating the risk of plant loss, be it technology or government schemes to increase the prospects of livelihood.

Eventualities of climate change have led to rethink-and-reorient the paradigm of food security through ‘more with less’ by harvesting every grain that has been cultivated in any region. This makes crop loss due to plant stress, whether biotic or abiotic, an important part of the food security challenge. Today, most of the flora is simultaneously subjected to both biotic and abiotic stress, leading to variation in the response pattern of the plants^12^. Therefore, to address this issue, it is important to understand the adaptation process adopted by plants to survive, when under stress. Researchers have reported enhanced uptake of macro-nutrients by plants under the stress of any type^13,14^. This makes the rate of uptake of nutrients from the growth medium/soil an important parameter to design a plant stress phenotyping system. Literature shows that Potassium (K) is one of the key elements which plays a vital role in plant response towards biotic or abiotic stressors^13^. It has been shown that crop quality as well as quantity has improved as the use of Potassium fertilizers increased in the crop field^15^. It has also been shown that Potassium fertilizers lead to the increased abiotic stress tolerance in the crops^16^. Under salt stress, K helps in maintaining the ion homeostasis and to regulate the osmotic balance. Under temperature stress, K may work as osmolyte and can help in maintaining stomatal conductance to prevent damage. The optimal amount of K in the soil also helps plants in mitigating the water deficit by using it efficiently^16,17^.

Identification of infestation or stress is generally carried out by farmers through visual symptoms on the plants, which occur after the crop has already suffered significant damage. To prevent losses, farmers use excessive pesticides which cause a domino effect giving rise to further problems such as environmental pollution by the chemicals used in pesticides, health issues to the farmer spraying pesticides in the crop field and poisoning of food grains by these chemicals^18^, which can lead to serious health conditions for consumers^19–21^.

A few prevalent stress detection techniques include thermography in which nutrient-dependent stomatal movements are visualized^22^, multi-spectral and hyper-spectral imaging in infrared and visible range^23,24^, and satellite and drone-based continuous monitoring techniques to keep track of plant growth and health over a wide agricultural area^25–29^, deep learning based plant stress phenotyping on digital images^30–32^. In the context of pest infestation, it has been reported that the extent of the same is related to temperature and humidity. These parameters have been used to qualitatively assess plant stress^33,34^.

These methods have proved to be reliable but are viable for large or large farm holders, due to the requirement of high computing power, extensive equipment and primarily depend on the appearance of specific visual symptoms of stress, which can vary for different stressors for different plant species^8^. For example, fungal disease shows chlorosis and leaf spots, whereas, bacterial disease shows leaf spots with yellow halo and fruit spots^35^. These symptoms can be seen when manifested, and by then the infestation is considerable and sometimes irreversible. Mostly techniques focus on biotic stress, however latent and yet potential stress causing agent is climate change and the response of plants and flora to these stressors is overlooked by farmers. It is important to understand that though climate change is an abiotic stress, it can potentially increase the magnitude of biotic stress in plants and crops^36^. Therefore, it is important that while addressing the issue of food security, plant health and sustainability should be considered than treating different probabilistic stress causing agents isolated. Additionally, these techniques do not consider the level of nutrients present in the soil, absorption rate of soil nutrients by the plants, and soil pH. Symptoms of plant stress may change with species and appear later. But, the rate of nutrient uptake changes significantly across all species because of the initiation of self-defence mechanism to survive, thrive and multiply days before manifestation of the symptoms on the plants^26^. Therefore, a method targeting change in rate of uptake of nutrients will be applicable to a larger variety of crops.

Advances in automated plant stress phenotyping systems mostly integrated with artificial intelligence (AI) are becoming the preferred choice of researchers as these techniques facilitate in dealing with large quantum of data arising from continuous monitoring of plants on a farm. Access to over-the-network computational power integrated with IoT sensors is key to high-throughput phenotyping and are providing a promising path forward to resolve challenges with plant stress phenotyping^37,38^. Here, we also use simple sensing systems augmented with machine learning algorithms to phenotype generic stress severity in plants. Both biotic and abiotic stress detection techniques with temperature and humidity as the primary parameters have been reported in the literature^33,34^. But, a high probability of fluctuation in the values of these parameters as a result of weather fluctuations can lead to unreliable results. Therefore, temperature and humidity cannot be treated as primary parameters for stress detection.

**Figure 1:**
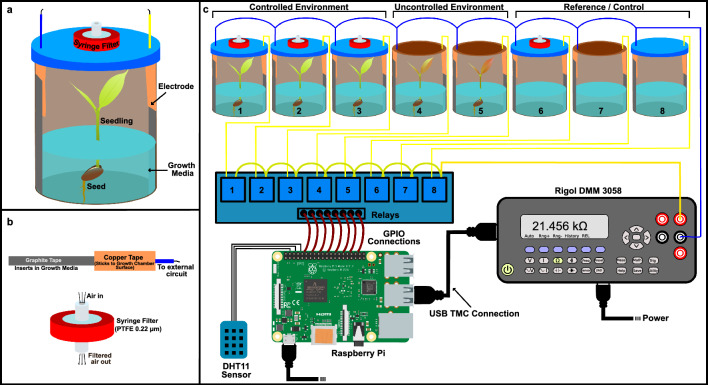
This figure shows the design of the experimental setup. (**a**) It shows the artificial growth chamber used to grow the plants and to measure the electrical resistance of growth media. (**b**) It shows the electrode made with graphite and copper tape and also the air filter used to provide clean air to the plants. (**c**) This figure is the representation of the final measurement setup consisting artificial growth chamber, relay modules to switch circuits, DHT11 sensor for measuring temperature and humidity, a digital multimeter for measuring the growth media resistance, with Raspberry Pi as controlling all the aforesaid components and storing data.

**Figure 2:**
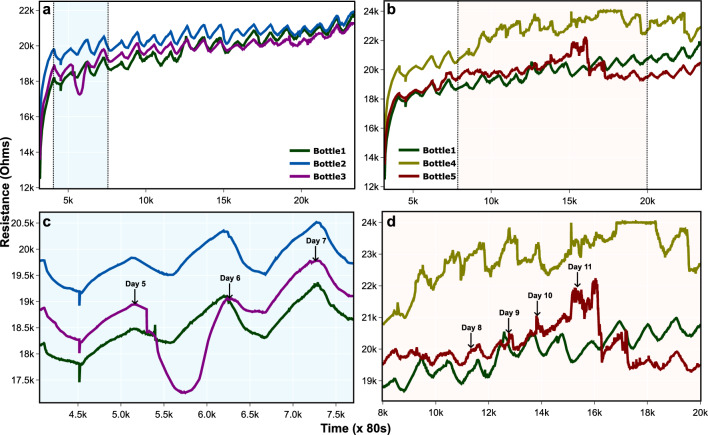
This figure contains the extracts from the original resistance data, with some key observations for the first experiment. For complete data, see Supplementary File Fig. S2. (**a**) Resistance curve of plants in bottles 1–3. (**b**) Resistance curve of plants in bottles 1, 4, and 5. (**c**) A sudden decline in the resistance characteristic of the plant in bottle 3 was observed on the 5$\text {th}$ day of the experiment. (**d**) Anomalous behaviour of the plants in bottles 4 and 5 can be seen from this figure for the respective days.

Motivated by the gaps and limitations in the literature and the phenotyping systems available, this paper proposes a novel stress sensing system, primarily taking variation in plant nutrient uptake from the soil to predict plant stress. The method is based on the continuous measurement of the earth electrical resistance of soil due to changes in the concentration gradient. Similar measurements are already used for precision agriculture^39^, applications in thermal imaging^22^, and quality evaluation of edibles^40^. The earth resistance values give an indirect measure of the soil nutrients, these nutrients act as the charge carriers for mediating the current flow. A high electrical resistance shows low current flow through the circuit, therefore, indicating a deficiency of charge carriers or nutrients. We hypothesized that when plants are stressed and show increased uptake of nutrients, then the change in growth media resistance will be directly proportional to the decrease in the number of charge carriers (see Eq. 1). This variation can be used to quantize the severity of the stress. To validate this, electrodes were directly placed into the growth media to also account for the short-lived variations in the nutrient uptake by the plants. Resistance was measured continuously for several days to get data at different physical conditions in the life cycle of the plants under study. The goal was to correlate variation in physical condition of the plant with the resistance pattern of the growth media. To minimize the noise in data, agarose was used for growing plants (Figs. 1, 2).

## Results

Two sets of experiments were conducted: iteration-1 was carried out for 20 days as proof-of-hypothesis testing and for optimizing the experimental design; iteration-2 was carried out for two months with a completely automated data collection system from the plant and surrounding environment.

### Iteration 1

In this set, data was observed for the possible variation in the electrical resistance of the growth media based on the physical condition and the stage of the plant in its life cycle (see Fig. 2). Experimental data showed a uniform increase in the resistance values with embedded periodicity (after applying linear fit), possibly indicating the variation in the environment during the day–night cycle. To analyze and verify any abnormality in the data, a linear fit was verified using the $R^2$ score (see Supplementary File Fig. S2) as shown in Fig. 4.

A continuous linear increase in the resistance of the growth media with an embedded periodicity of increase and decrease was observed. The overall increase in resistance of the growth medium, which can be considered a highly concentrated ionic suspension, is due to the decrease in net ionic content (macro-nutrients) of the growth medium that has been taken up by the plant over the entire time window. The embedded periodicity appears very close to the diurnal cycle of plants, but the affecting parameters here should be both exogenous and endogenous. In the daytime, when the temperature rises, the electrical resistance of the growth media decreases, and at the night, when the temperature falls, the resistance of the growth media increases (Eq. 1). A divergence from the observed overall increase in resistance can be an indicator of abnormality or stress. It has been reported that plants take more macro-nutrients, in particular $K^+$ ions, while being stressed^13^. Intake of these cations creates a scarcity of charge carriers in the growth media, resulting in an increased rate of change of resistance in those instances as compared to the overall trend. Plants, when stressed, release allelochemicals: a chemical trigger of stress to other plants through the root network, in the soil. This can increase the charge-carrier concentration in the growth media, therefore, decreasing the resistance drastically^41,42^. The resistance curve of bottle 5 showed a large divergence from linear fit and a low $R^2$ score, indicating maximum severity of stress. But resistance data of the plant in bottle 5 came closer to that of bottle 1, indicating the ability to fight the adversity of stress and survive. The linear fit analysis gave a threshold $R^2$ value (0.65), below which the plant is most likely to be stressed in iteration 1. This experimental analysis clearly indicated the role of charge carrier concentration of the nutrient media to be strongly correlated to the onset of stress in plants. It was also clear that a robust/reliant analysis should take into consideration appropriate environmental data for accurate prediction (e.g. water table and scourge of stubble burning).

#### Automated stress phenotyping for Iteration-1

**Figure 3:**
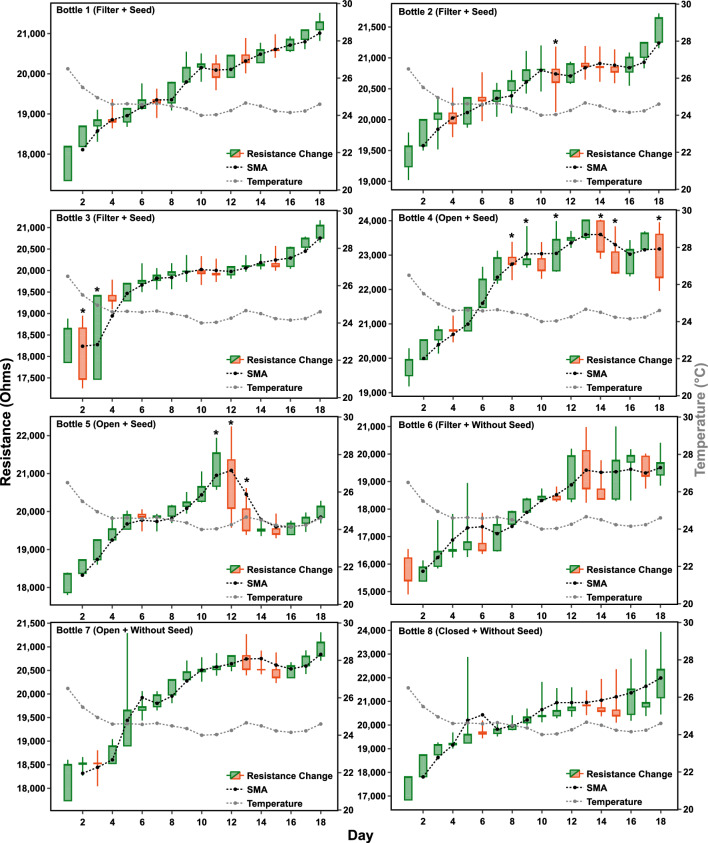
The candlestick trace shows the variation of the resistance. The black dashed line shows the simple moving average (SMA) of the resistance, which shows the overall trend of the resistance. The gray dashed line shows the variation of average temperature for the period of the experiment. In these graphs, a large difference between low and high values (indicated by the * sign) of resistance means abnormal behaviour of plants that can be attributed to stress. The stress leads to higher uptake of the nutrients, therefore, increasing the electrical resistance (see Supplementary Eq. A1 in Appendix A.pdf). For raw graphs, see Supplementary File Fig. S2.

**Figure 4:**
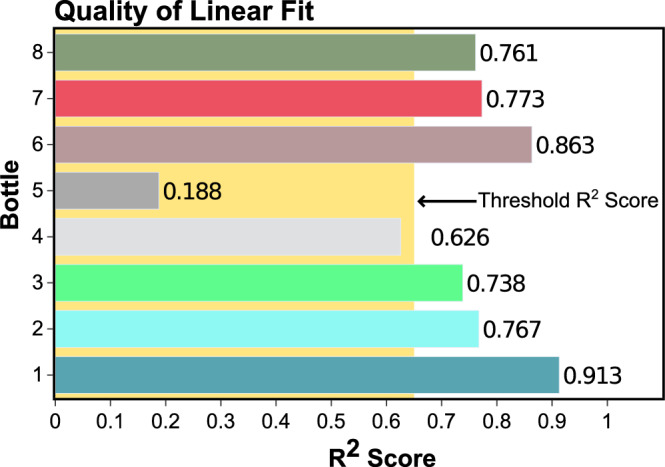
The above figure shows the quality of the linear fit of resistance characteristic of all the bottles using $R^2$ test for the first experiment.

The enormity of data arising from the continuous monitoring of plants opens the realm of machine learning and deep learning. However, identifying the right method and algorithms becomes crucial in the sensitivity and accuracy of forecasting. These learning patterns are recalled to identify anomalies or outliers in live data coming from the real-time system. While analyzing the data in this work, we took an explorative trajectory from various machine learning algorithms to deep learning techniques. Initially, data analysis was carried out with a basic premise that data is in abundance, but anomalies are rare. Analysis of the data was carried out using three machine learning algorithms; k-Nearest Neighbour (k-NN), Local Outlier Factor (LOF), and One-Class Support Vector Machine (OCSVM) in an unsupervised learning mode as the anomaly labels are not available^43^. To create an autonomous stress phenotyping in plants, anomaly/outlier detection, and machine learning method was applied to the electrical resistance characteristic of the growth media^43^ to produce a decision score based on which the severity of plant stress was measured (i.e. high score, high stress) (see Fig. 6). The results generated from the above algorithms were compared to find out the most suitable algorithm based on sensitivity, identification of stress, and resolving power (ability to distinguish between stressed and non-stressed/default stressed conditions). In our observations, LOF could not distinguish between stressed and non-stressed resistance characteristics, as it was showing the same decision score for stressed and non-stressed cases in a combined characteristic graph. The OCSVM technique was too sensitive and showed a high decision score on even minor fluctuations in the resistance. Finally, k-NN produced close to 0 decision score in non-stressed condition and close to 40 for stressed condition (see Fig. 6). It could distinguish stressed and non-stressed conditions effectively (see Table 1). Therefore, it was taken as primary stress predictor for the anomaly detection software (see Supplementary Fig. S3). A live stress tracking software based on anomaly detection (see Supplementary Video File Anomaly Detection.mp4 and Supplementary Fig. S3) was created, which produces a live decision score on the continuous time series measurement of resistance of the growth medium. This can act as a trigger to set an alarm for the farmers. The results of the algorithms were compared, and it was found that the kNN algorithm works best as an anomaly detector on our data. This method has been filed for a patent in the Indian patent office (published with application number 202111033265, published in 2021)^44^ (Figs. 3, 4).

**Table 1:** The table shows the comparison between the algorithm used to predict plant stress for Experiment 1.

Feature	Algorithm		
	kNN	LOF	OCSVM
Sensitivity	Moderate	Low	High
False score	Low	Moderate	High
Resolving power	High	Low	Low

### Iteration 2 of the experiment

From iteration 1 of the experiment, it was clear that the electrical resistance of the growth media significantly changes when plants are stressed. Therefore, it can be taken as an important parameter to predict plant stress. However, the resistance of the growth media was also affected by the diurnal changes in the environment. Therefore, effect of the environment is also included in the iteration 2. Drude’s model for ionic solutions is used for analysing the effect of temperature on the resistance of growth media and relative variation of charge carriers^45^. For prediction of stress, anomaly detection was applied to the resistance measurements as a time series data and results from different algorithms were compared.

#### Calculation of relative change in charge carrier concentration by resistance measurements

The dependence of resistance on temperature (*T*) and charge carrier concentration (*n*) due to temperature compensation factor ($\alpha$) can be given by Supplementary Eq. A3 (see Supplementary Appendix A). Here, $T_0$ is the reference temperature.

1 $$\begin{aligned} R \propto \frac{1}{n[1 + \alpha (T - T_0)].} \end{aligned}$$

The above equation accompanied by Drude model (Supplementary Eq. A1 Appendix A^45^) can be used to calculate the relative change in the charge carrier concentration of the growth media for temperature compensation factor $\alpha = 0.0141/^\circ$C^46^ (see Supplementary Tables S1 and S2 for calculations). The final equation is given by (see Supplementary Appendix A for complete derivation),

2 $$\begin{aligned} \frac{n - n_0}{n_0} = \frac{R_0}{R[1 + 0.0141(T - T_0)]} - 1. \end{aligned}$$

The plant in bottle 2 (any plant can be chosen) was used for calculating the change in charge carrier concentration, where the reference temperature and the resistance were $T_0 = 14.98^\circ$C and, $R_0 = 22015\Omega$ respectively. These results were compared with already published results^41,42^ and were found to be in good agreement with them.

It was observed that the variation of charge concentration was 5% when plants were not stressed. Therefore, to change this behaviour, plants were stressed by pouring soil in the bottles and plate count agar was used to find out the microbes present in the soil (see Supplementary File Fig. S6)^47^. It was observed that the relative change in charge carrier concentration increased from 5% (see Fig. 7b) to 35% (see Fig. 7d) after 3 days of pouring the soil.

#### Automated stress phenotyping for Iteration-2


**Figure 5:**
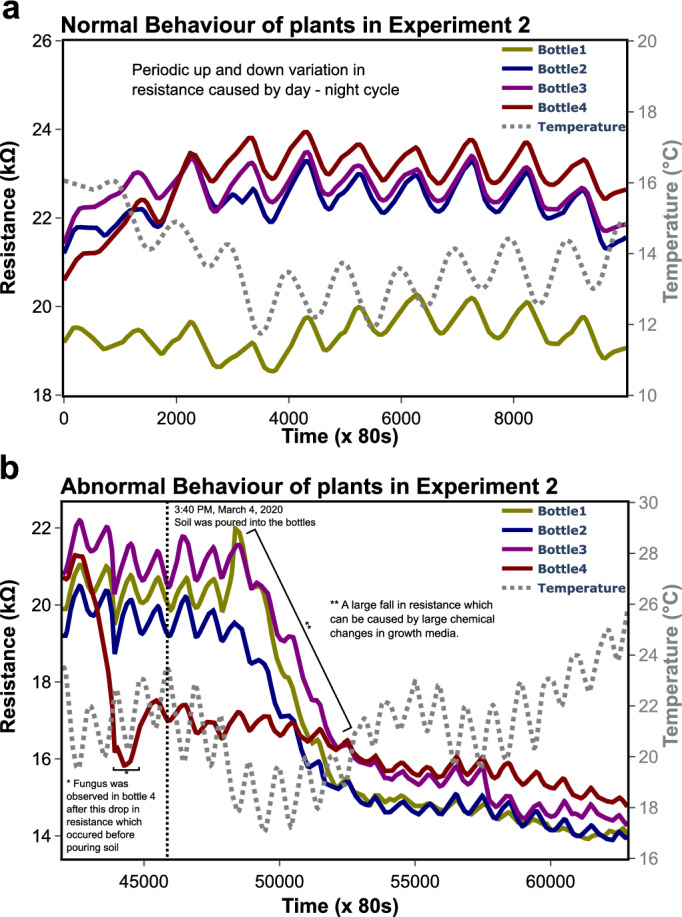
These graphs are extracted from the original data collected over a period of two months from the second experiment. (**a**) It shows a non-stressed or normal pattern, showing the repeatability with the first run of the experiment. Diurnal effects can also be observed from the graph. (**b**) We dropped some soil (on March 4th, 2020) in all the bottles except 8 to see if that would change the plant response. After three days, a sudden decline in resistance was observed for all the plants except plant 4.

**Figure 6:**
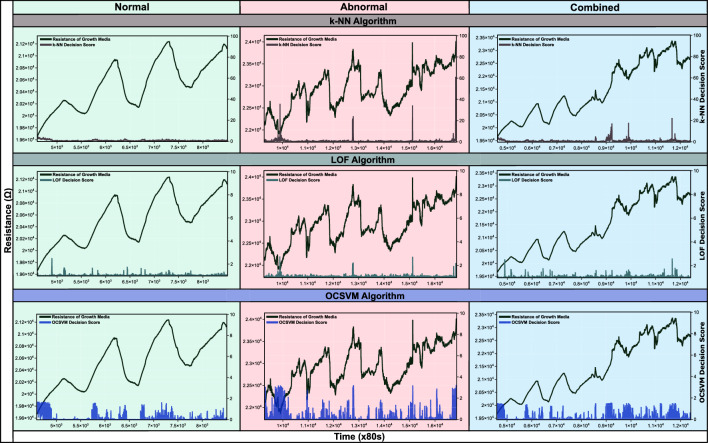
The graph shows the comparison of decision scores between k nearest neighbour classifier (kNN), local outlier factor (LOF) and one-class support vector machine (OCSVM) for outlier detection on the growth media resistance data of plant 4 for experiment 1. Normal condition shows the data for day 1–3, abnormal condition shows the data of day 5–11, and combined data shows the variation in resistance from day 1 to 8.

**Figure 7:**
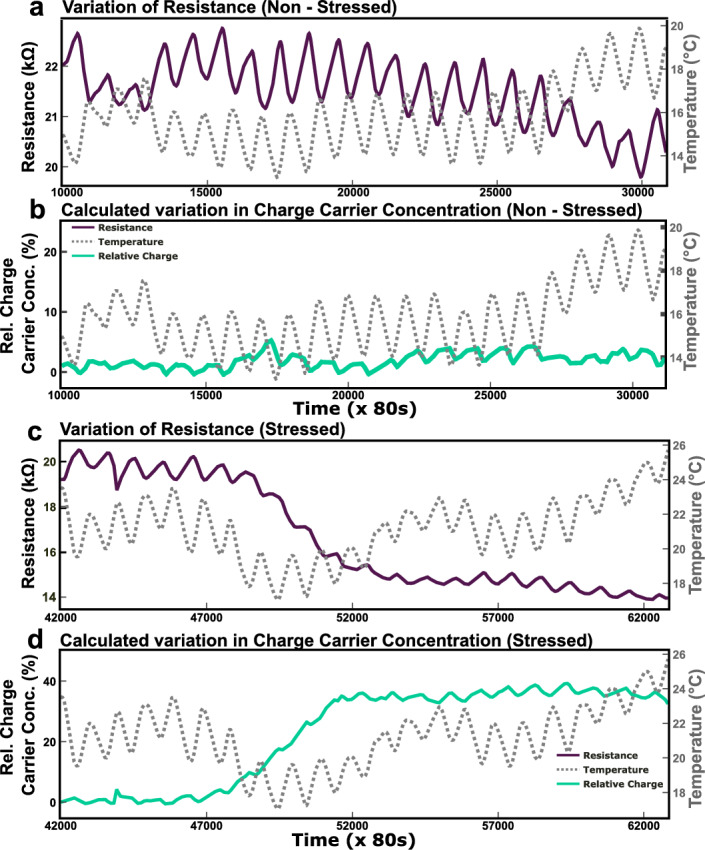
This figure shows the variation of resistance and the calculated change in charge carrier concentration before and after artificially inducing stress by putting the soil in the bottles. (**a**) It shows the variation of resistance in non-stressed condition. (**b**) It shows the calculated variation of the charge carrier concentration in the non-stressed condition. (**c**) It shows the variation of resistance in stressed condition of the plant. (**d**) It shows the calculated variation of the charge carrier concentration in stressed condition, i.e. after putting the soil.

**Figure 8:**
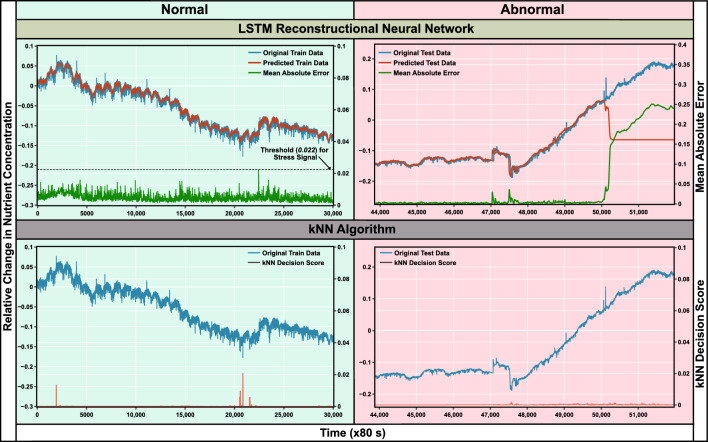
The figure shows the comparison between the anomaly scores generated by kNN algorithm and Long Short-Term Memory (LSTM) reconstructional Neural Network in both normal and abnormal conditions. The results generated show that LSTM neural network is much superior technique over kNN algorithm for predicting the anomaly in the data of experiment 2.

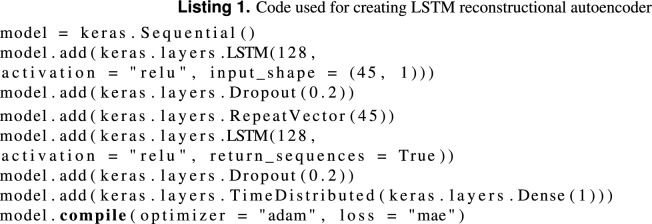
 (Figs. 5, 6, 7 and 8).

In iteration 2, immediate climatic conditions were incorporated by measuring temperature and humidity. The Drude model as described in the previous section was used to analyze the change in charge carrier concentration of the growth media due to the uptake of nutrients by plants. Measurements were carried out on the same Bengal gram seeds for 2 months. The experimental setup was the same as before, with measurements of environmental parameters also included. However, the machine learning anomaly detection algorithms used in the iteration-1 data did not work effectively on the iteration-2 data set. kNN algorithm was giving false decision scores, therefore, the deep learning method, based on artificial neural network(ANN) was considered. After training the data set on several options, Long-short Term Memory (LSTM) reconstructional autoencoder was considered (see Listing 1, Supplementary Fig. S7). For training and testing the LSTM model, different input vector sizes were used, finally, (45, 1) was utilized to train the model. Results generated by the ANN were compared with the previously used kNN algorithm (see Fig. 8). The LSTM network was trained on the data set for 30 days, which had very scarce anomalies. After training the neural network on the training dataset, the maximum reconstructional error was taken as a threshold for predicting the anomaly (see Fig. 8). This data set from Day-1 to Day-30 is called Training Data, and from Day-31 to Day-60 is considered as ‘Test-Data’. Test-Data showed anomalies indicating stress, the severity of stress was calculated based on the reconstructional error that the model generated, called Mean Absolute Error (MAE)). On the training data, the maximum MAE value was 0.022 (see Fig. 8), and therefore, it was taken as the threshold to differentiate between default stress and the induced biotic and/or abiotic stress in the plants. It is to be noted that the decision score of kNN on the training data was almost equal to the decision score generated by LSTM, however, kNN was inefficient in resolving anomalies in the Test Data and produced close to 0 decision score. On reconstructing the test data using a neural network, it was observed that after 50,000 data points in the Test Data (i.e. after the 49th day of iteration-2), MAE jumped to 0.25 indicating an anomaly in the Test Data and the reconstructed variation in nutrient concentration became constant. As the neural network was not trained for tolerance of 10% for the anomalies in the Test-Data, any change more than the tolerance limit is considered by LSTM as an unlearnt pattern and error including score was generated by reconstructing the difference from the values generated in Training Data.

## Discussion

Every living system is under constant latent stress to survive and thrive/sustain. Therefore, it was important that the analysis led to the identification of stress in plants due to external causes, whether biotic or abiotic and was able to resolve it from the latent default stress that every living system undergoes as a process of surviving. Precision agriculture has greatly benefitted from technology and techniques like IoT and automated anomaly detection in managing, and monitoring plant health^48^. In iteration 1, resistance measurements were carried out, which was the direct measure of the nutrient uptake from the growth media. The nested diurnal variations were also observed, indicating the effect of 24 h cyclic change in the immediate ecology of the plants (Supplementary File Fig. S2). However, for a few plants, divergence from the linear fit was observed. For these graphs, the $R^2$ score was calculated to see the quality of the linear fit. It was observed that the measured resistance was highest in the morning (7 a.m.–8 a.m.) and lowest in the evening (4 p.m.–5 p.m.). Agarose shows such a response because of its ionic nature, which leads to a temperature compensation factor^46^. Bottles 1–3 were kept with air filters, and the resistance curve of growth media in these bottles showed a periodic up-and-down movement. A decline was observed in resistance in bottle 3 on the 5${\text {th}}$ day of experiment 1 (see Fig. 2a,c) which indicated it was stressed, and it was confirmed by fungal growth on visual inspection in bottle 3 the next day (see Supplementary Fig. S4). This decline is due to an increase in the charge carrier concentration caused by gram seed releasing allelochemicals in the growth media^49^. The $R^2$ score of linear fit for plants 1 to 3 was 0.913, 0.767, and 0.738 respectively. Bottles 4 and 5 were without air filters and started with a response similar to bottles 1–3. However, after 8 days, the response of both the bottles became noisy (see Fig. 2b,d) with a large variation in the resistance of bottle 5 on 12$\text {th}$ day of the experiment (see Fig. 2b and Supplementary File Fig. S2). The $R^2$ score of linear fit was 0.626 and 0.188 for bottles 4 and 5. The $R^2$ scores for bottles 6, 7 and 8 were 0.863, 0.773, and 0.761, respectively. We were particularly interested in the daily variation of the resistance that could be assessed from the large amount of data collected. Therefore, this data was visualized with a candlestick chart providing all the necessary information (high, low, open, and close resistance of the day). This data was also analysed with anomaly detection using machine learning. Plant stress detection was based on the decision score generated by k-Nearest Neighbour (kNN) algorithm.

**Figure 9:**
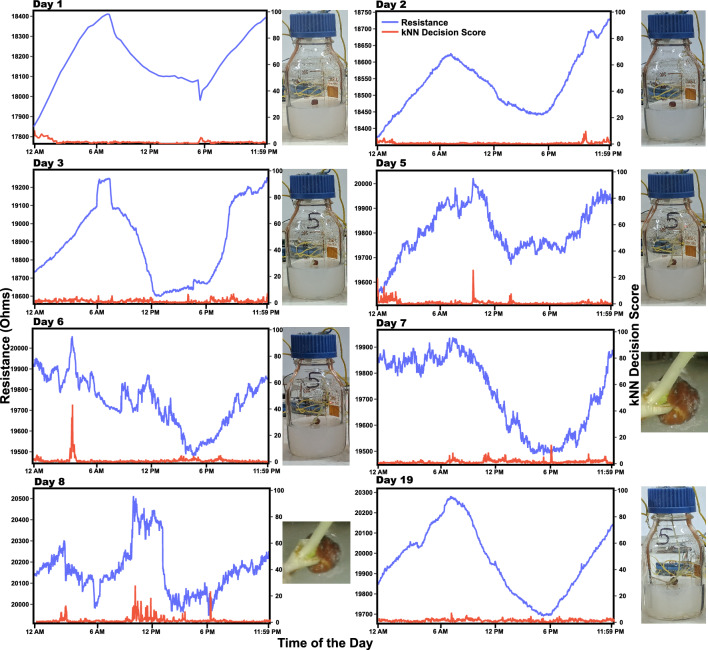
The figure shows the day-wise variation of resistance of plant 5 and calculated decision score by kNN algorithm and the physical condition of the plant showing if it is stressed or not stressed. It can be seen that the plant starts to get stressed on day 5 (evident from resistance data and kNN decision score), however, actual symptoms are seen after two days. Further, the decision score goes close to 0 on 20${\text {th}}$ day of the experiment, meaning the plant could fight stress and overcome it.

The performance of an algorithm and its prediction can only be verified through validations and claims. Data from plant 5 was chosen to validate the predictions of kNN algorithm. Data is analysed day-wise in Fig. 9 with 24 h time series for each day starting from 12:00 a.m. Midnight to 11:59 p.m. Changes in the resistance data from Day 6 to Day 9 clearly indicate patterns of stress, while the visual symptoms appeared on the plant on Day 10. When the plant is not stressed, the decision score generated by the kNN algorithm remains below 20, whereas, when under stress, the decision score jumps to 40. A closer look at the resistance data of plant 5 revealed a change in the diurnal pattern of resistance when the plant just began to experience stress. During normal conditions, the resistance steadily increased from midnight to dawn every day and thereafter decreased from around dawn to dusk. This pattern showed a change (see Fig. 9 Day-6 to Day-11 data). Interestingly, a visual effect on the plant corresponding to the a priori change in the resistance data was seen only on Day-11. Here fungus growth on the plants is seen, which is a stressor itself (see Day-11, Fig. 9). This clearly indicates the sensitivity of plants to changes in their immediate ecology and a natural tendency to overcome them (Day-20). Analysing the resistance data for longer periods reveals that plants like any living system fight the impact of stress and if the stressor is not potential enough then plants come out as winners! As seen in the resistance data of Day 20. Towards the end of the experiment, the decision score was close to 0 and good growth was observed for the plant. From the automation of stress forecasting for iteration-2 data, it emerged that ANN-based deep learning methods were more efficient and accurate in forecasting stress using multiple parameters.

Similar diurnal patterns were observed with no visible signs of stress either in the time-dependent gradient in the charge carrier concentration or on the plant itself (Fig. 5a). To perturb the homeostasis of plant, soil was added. Figure 7 shows the experimental simulation of stress which was not seen on the plant. Here, soil from the exposed plant bed outside the lab was considered as the stress-phantom to stimulate stress by introducing it in the growth media^47^. Agar plating of the soil was carried out and the presence of *Serratia marcescens* was identified (the orange colony in Supplementary Fig. S6). Strains of *S. marcescens* are known to have many pathogenicity and virulence factors, however, it is not known which virulence factor is phytopathogenic. After three days, a sudden decline in resistance was observed (Fig. 5b) for all the bottles. This behavior was confirmed as stress when fungus growth on the plant in bottle 4 was observed after some days (which means it was already dead by the time soil was put in the bottles, hence didn’t show any response). The decline in resistance was helpful in calculating the change in charge carrier concentration by applying the modified Drude model. This change in charge carrier concentration indicates the number of allelochemicals released in the media or the nutrients absorbed by the plants, which in turn is indicative of the severity of plant stress. Figure 7a,c shows a change in resistance for the plant in bottle 2. It is interesting to note that under normal conditions, temperature-dependent resistance change was ± 1 kOhms, when under stress resistance did not follow the temperature dependence and the change was six times that of normal conditions as seen in Fig. 7c. Figure 7b and d show the relative percentage change in the charge carrier concentration of the growth media calculated from the change in resistance. It can be seen that under normal conditions relative percentage change is within 5 percent and under stressed conditions there is a sharp increase to 35 percent. This indicates an increase in ionic content of the growth media due to the release of large amounts of allelochemicals by the root network to send warning signals to the neighbouring plants as a default physiological reaction to counter stress. This change was compared with the already reported values of the relative change of charge carrier concentration in growth media when plants are stressed^42^ which was close to 33% relative change. This validates our experimental procedure. However, this multivariable problem of plant stress, food security and climatic change require an abstract topological approach where the artificial general intelligence of all the stakeholders is invoked through conscientiousness and consciousness with power law for the simulation of basis vectors of land space for a sustainable healthcare habitat^50^.

## Conclusion

The studies conducted in this paper bring out that plant stress, whether biotic or abiotic, can be either in manifest or unmanifest form. The manifest form of stress is seen on the plant as visual symptoms due to infestation, like in this paper the growth of fungus in iteration-1. Unmanifest plant stress mostly goes unnoticed as its effect is either not seen on the plant, or if observed, is at a later stage, when the physiological functions of a plant asymptotically fall.

From the electrical measurements carried out in this work and its detailed analysis, it emerges that there are two types of stress-causing factors: local, which can be due to infestation, and alteration in the growth media (soil) conditions, and global, which is caused due to climate change leading to extreme weather conditions (Nature with infinite variety) and thus affecting the local stress causing factors. A study has reported that with every rise of 2 $^\circ$C in global surface temperature, pest infestation (biotic stress) increases by an average of 32%^36^. Therefore, it becomes pertinent that when food security and crop loss have taken the center stage in the UN sustainability initiatives for the planet Earth, the problem at hand is addressed just not from the viewpoint of agriculture but from the agroecological point. This paper presents a scalable, completely automated through ANN and yet frugal technique for a priori forecasting of multiplexed stress (both local and global) in plants. This technique will be of potential use for farmers with small holdings who cater to the demands of small communities around them and are generally impacted most by the local and global stress on their crops. It was observed that when plants are under stress, there is a large variation in resistance, which is significantly different from its regular periodic variation during the day-night cycle. This change in resistance of growth media during stress leads to a change in nutrient concentration by 35% which is in agreement with previously published studies^41^.

## Methods

Bengal gram (*Cicer arietinum*) plants release large amount of allelochemicals through the root network when under stress^41,51^ were grown in the lab conditions for the study. These seeds were provided ambient daylight, water was provided with the growth media, and in the experimental iterations, the temperature was not controlled. The study complies with local and national guidelines for the experimentation on plants. The effect of stress was measured under different environmental conditions (controlled and uncontrolled). Experiments were conducted twice on independent sets of plants. In the first experimental iteration, only electrical resistance was measured. The effect of external environmental parameters was taken into account in the second set of experiments by measuring temperature and humidity. Parameters were measured by data collection hub comprising Rigol Digital Multimeter model 3058 (Rigol DMM) for resistance measurement, a module of 8 relays (for 8 bottles see Fig. 1a) for switching to different plants (see Fig. 1c), DHT11 sensor for measuring temperature and humidity (in the second experiment) and a Raspberry Pi model 3B (RPi3B) for controlling all other instruments and saving the collected data (resistance, temperature, and humidity) in its local memory card (see Fig. 1c). Measurements were made sequentially on all the eight plants at an interval of 10 s. 8 plants were grouped in three categories based on the surrounding environmental conditions as controlled, uncontrolled and reference (see Table 2). Uncontrolled environmental conditions were given primarily to understand stress pattern in plants, resulting in random intake of macro-nutrients such as potassium and release of allelochemicals in variable quantities^13,41,42^.

**Table 2:** The arrangement of the bottles for including different conditions for the plants.

Bottle	Setup	Type
Bottle 1	Seed + with air filter	Controlled environment (Nurture/Sustainable)
Bottle 2		
Bottle 3		
Bottle 4	Seed + without air filter	Uncontrolled environment (Nurture/Sustainable)
Bottle 5		
Bottle 6	Without seed + With air filter	Reference/control
Bottle 7	Without seed + Without air filter	
Bottle 8	With agarose and closed	

Borosilicate bottles were converted to a frugal yet ingenious resistance measurement setup by introducing graphite tape as electrodes and copper tape as a point of contact for the external circuit (see Fig. 1b, Supplementary Fig. S1A). Agarose solution (conc. 8 g/L (wt/vol)^52^) in distilled water was heated to melt and dissolve the agarose, and 100 mL of the same was carefully poured into all 8 bottles. These bottles were sterilized by autoclaving at 15 psi pressure and 125 $^\circ$C temperature for 20 min. Bengal gram seeds were then dropped in the first five bottles and air filters were placed as mentioned in Table 2. Rigol DMM was connected to RPi3B on usb-tmc via USB connection. A relay module was connected to GPIO pins of RPi3B to initiate the experiment (see Fig. 1). The first iteration of the experiment ran for 20 days and more than 20,000 data points were collected from each bottle. The second set of experiments was conducted for 2 months and, 62,800 measurements were taken on each plant which included the measurement of temperature and humidity to account for stress, if any, due to climatic conditions. In iteration 1, a linear fit model was applied to the collected data and the $R^2$ score was calculated to observe the divergence from the linear fit for each plant. Divergence from the linear fit is an indicator of the level(s) of plant stress because plants increase the uptake of the nutrients from the growth media when stressed, which leads to the increase in electrical resistance^13,49^ (Supplementary Eq. A1). Data was also visualized using candlestick charts to observe day-to-day variation in resistance. Candlestick charts are conventionally used to forecast stock value change^53^ (see Supplementary text). We adopted candlestick charts for representing a change in resistance patterns. The visualization of resistance with candlesticks forecasts the onset of stress signal (see Fig. 3). In experiment 2, the Drude model was applied to understand the phenomenological variation in charge carrier concentrations under stressed and non-stressed conditions. This calculated variation was compared with reported values for verification of the study^41,42^.

## Supplementary Information

Supplementary Information 1.

Supplementary Information 2.

Supplementary Information 3.

## Data Availability

Data is available upon request. Requests can be made to Mridul Kumar.

## Code availability

Available upon request. Requests can be made to Mridul Kumar (mridulsharma@dei.ac.in).
