# Supplementary material for: Decoding the physiological response of plants to stress using deep learning for forecasting crop loss due to abiotic, biotic, and climatic variables
Source: Sci Rep. 2023 May 26;13:8598. doi: 10.1038/s41598-023-35285-3 (PMC10215062; doi:10.1038/s41598-023-35285-3)
Supplement: Supplementary file 1 — Supplementary Information 1. [file 41598_2023_35285_MOESM1_ESM.pdf]

Decoding the Physiological Response of Plants to Stress using Deep Learning for Forecasting Crop Loss due to Abiotic, Biotic, and Climatic Variables

Scientific Reports

Mridul Kumar[1], Zeeshan Saifi[1], and Soami Daya Krishnananda[1]

[1] Microwave Physics Lab, Department of Physics and Computer Science, Dayalbagh Educational Institute, Dayalbagh, Agra, 282005, Uttar Pradesh, India

Corresponding Author : Prof. Soami Daya Krishnananda (ksdaya@dei.ac.in)

Supplementary Information Text

Candlestick Charts

Invented in early 1700s by Japanese rice trader Sokyu Honma candlesticks charts have been around for a very long time for tracking price of commodities (1). In present time these charts are heavily used in stock market for visualizing share prices. A single block of candlestick chart shows the variation of price for a period of time (5 minutes, 1 hour etc.). It has two parts, a body (red or green part) and a wick (line coming out of body). The top of wick shows the highest price whereas the bottom of wick shows the lowest price of the share for some time period. Candlesticks are of two types bullish (green) and bearish (red) (see Fig S2). Depending on the type of the candlestick the top and bottom of candle shows the open and close price of that time period. In the case of green candle the bottom shows open price and top shows closing price, but, the opposite is true for a red candlestick.

In our case, one candlestick represented the data for one day. In every candlestick the open was taken to be the first reading after or on 12:00:00 AM and the close was taken to be the last reading before or on 11:59:59 AM. The high and low were the highest and lowest resistance values for the day respectively.

Calculation for the temperature compensation factor

The temperature compensation factor (α) was calculated by extracting the highest and lowest temperature and resistance values from the resistance graph of plant 1 in second iteration of the experiment and putting those values in the equation $\alpha = \frac{R_{ref} - R}{R(T - T_{ref})}$ (2). The value of α was calculated in two cases. First when the resistance was rising and temperature was falling and second when the resistance was falling and temperature was rising (See Table S1 and S2). The mean of all the alpha values was calculated which gave us the final temperature compensation factor ($\alpha = 0.0141/°C$) that we used for calculating the variation in charge carrier concentration in the media in our second experiment.

Table S1: Resistance and temperature values extracted from the resistance graphs when the resistance is increasing in the night cycle.

**Rising Resistance and Falling Temperature**				
$R(\Omega)$	$T(°C)$	$R_{ref}(\Omega)$	$T_{ref}(°C)$	$\alpha=\frac{R_{ref}-R}{R(T-T_{ref})}/°C$
19827	14.15	18970	17.57	0.0126
20132	14	19344	16	0.0196
20368	13.44	19593	15.9	0.0154
20729	13.36	19741	16.11	0.0173
21490	13	20329	15.81	0.0192
21667	13.25	20727	15.9	0.0163
21749	13.9	20747	16.57	0.0173
21732	14	20708	16.9	0.0162
21901	14	21154	16.8	0.0122
22175	14	21262	16.8	0.0147
**Mean**				**0.0161**

Table S2: Resistance and temperature values extracted from the resistance graph when the resistance is falling in day cycle.

**Falling Resistance and Rising Temperature**				
$R(\Omega)$	$T(°C)$	$R_{ref}(\Omega)$	$T_{ref}(°C)$	$\alpha=\frac{R_{ref}-R}{R(T-T_{ref})}/°C$
19344	16	19827	14.15	0.0135
19593	15.9	20132	14	0.0145
19741	16.11	20368	13.44	0.0119
20329	15.81	20729	13.36	0.0080
20727	15.9	21490	13	0.0127
20747	16.57	21667	13.25	0.0134
20708	16.9	21749	13.9	0.0168
21154	16.8	21732	14	0.0098
21262	16.8	21901	14	0.0107
21484	16.9	22175	14	0.0111
**Mean**				**0.0122**

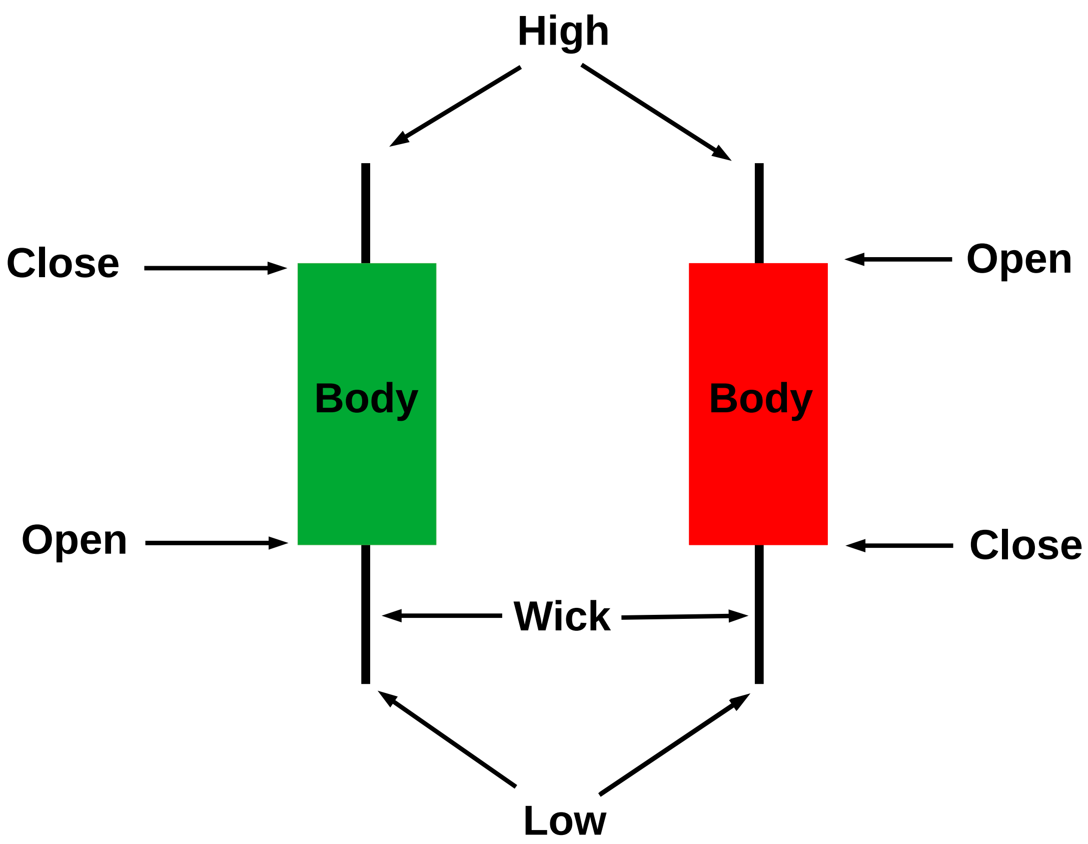

Figure S1 : Candlestick charts are used for visualizing price trends of stocks. A single candlestick represents the price variation in a time period (e.g. 5 mins, 10 mins, 1 hour) by visualizing four price values, open, close, low and high. It has two parts body and wick. Body represents open and close prices, and wick represents lowest and highest values of that period. Candlesticks are of two types based on open < close (green and bullish) and open > close (red and bearish).

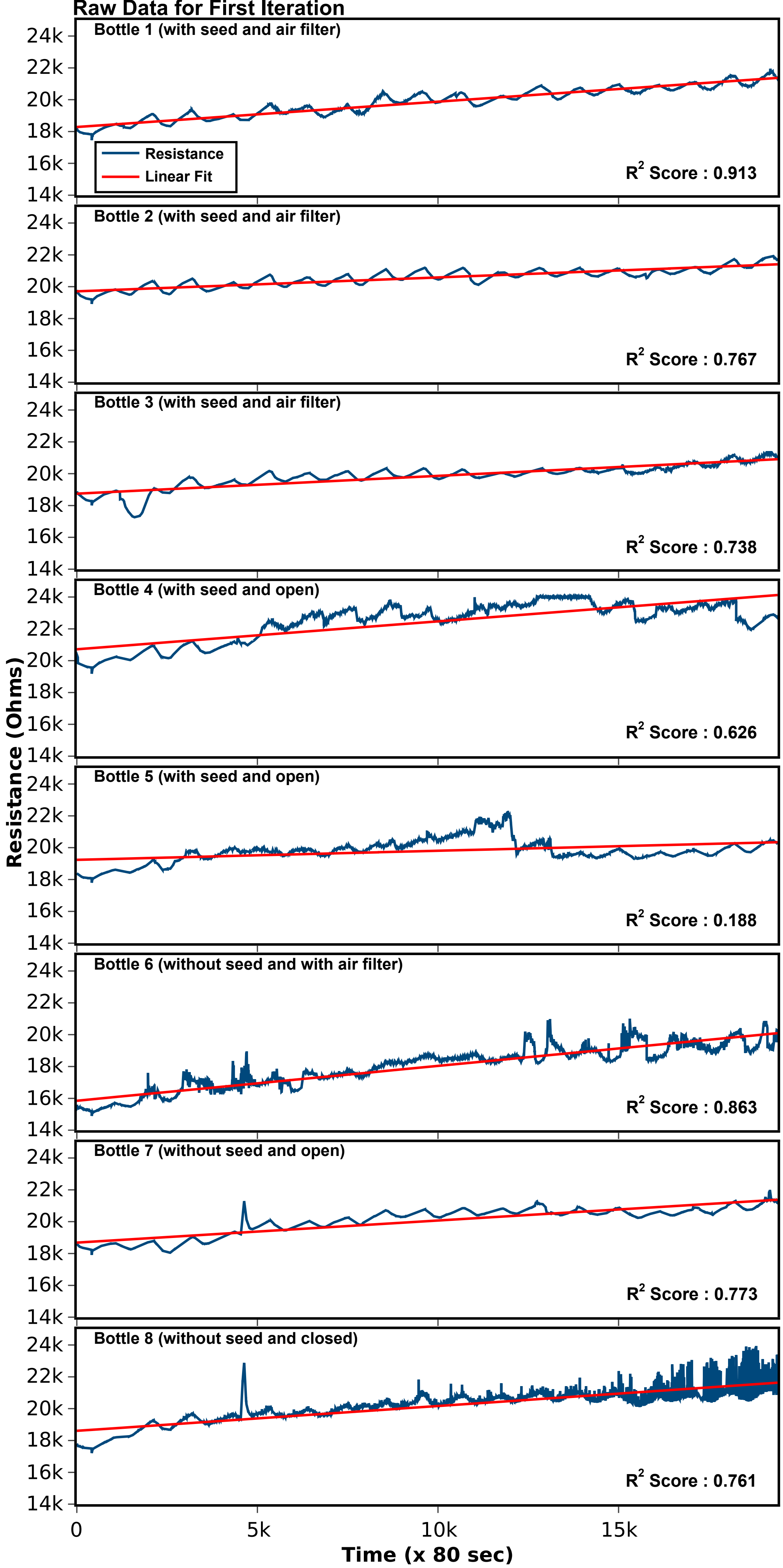
Raw Data for First Iteration
Bottle 1 (with seed and air filter)
Resistance
Linear Fit
R² Score : 0.913
Bottle 2 (with seed and air filter)
R² Score : 0.767
Bottle 3 (with seed and air filter)
R² Score : 0.738
Bottle 4 (with seed and open)
R² Score : 0.626
Bottle 5 (with seed and open)
R² Score : 0.188
Bottle 6 (without seed and with air filter)
R² Score : 0.863
Bottle 7 (without seed and open)
R² Score : 0.773
Bottle 8 (without seed and closed)
R² Score : 0.761
Resistance (Ohms)
24k
22k
20k
18k
16k
14k
0
5k
10k
15k
Time (x 80 sec)

Figure S2. Raw data for experiment 1 with linear fitting and R^2 scores. A higher R^2 score can be interpreted as good health for the plants and vice versa.

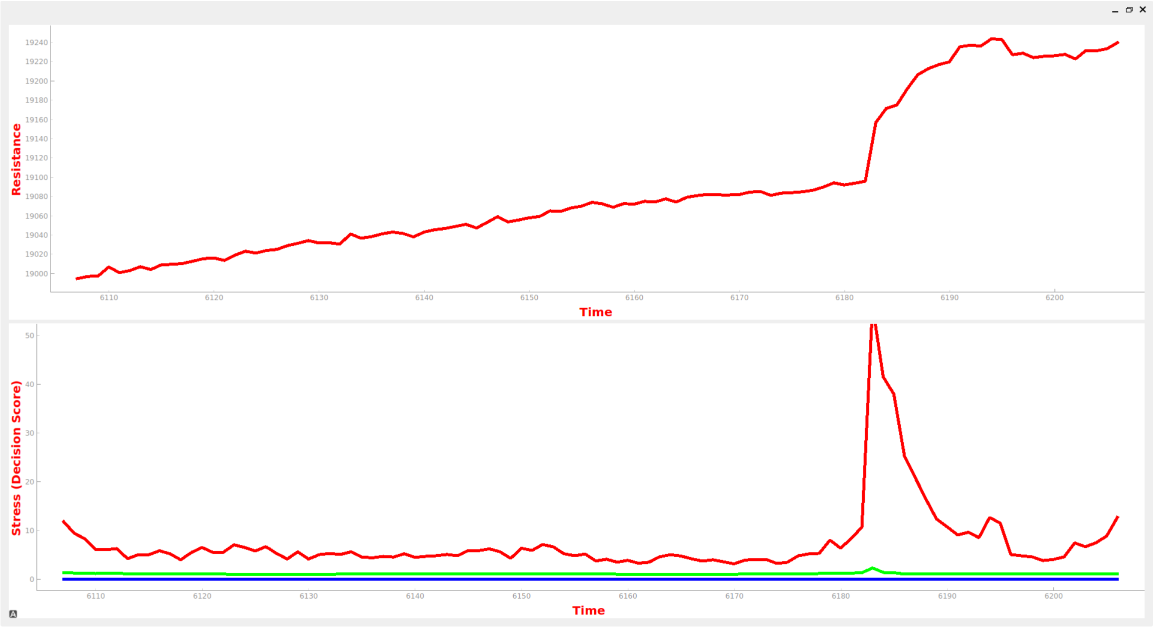

Figure S3. This figure shows the anomaly detection algorithm at work. The top graph shows the resistance data collected from plant and the bottom graph shows the decision score calculated by k – Nearest Neighbor (k-NN in red), Local Outlier Factor (LOF in green) and One-Class Support Vector Machine (OCSVM in blue) algorithms (3). A high decision score means an anomalous behavior is being shown by the plant that could indicate stress. However, for a normal behavior a very low decision score is seen.

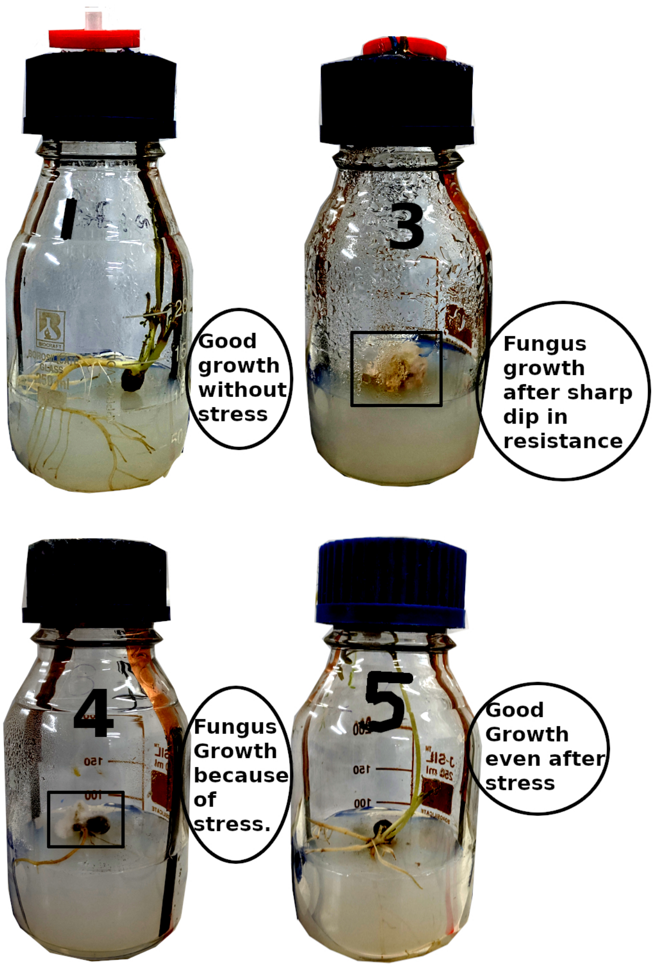

Figure S4. This figure shows the plants after the completion of first experiment. It can be observed that first plant didn't show any fungus growth and its resistance characteristic didn't show any anomalies either, 3rd plant showed a decline in resistance and after that fungus growth could be seen on it, 4th plant showed a anomalous behavior in the middle of the experiment and later got the fungus growth. On the other hand, 5th plant showed an anomalous behavior in between the experiment, but, later on it again became normal meaning it could fight off the stressors which can be confirmed from its growth which was the highest among all the plants.

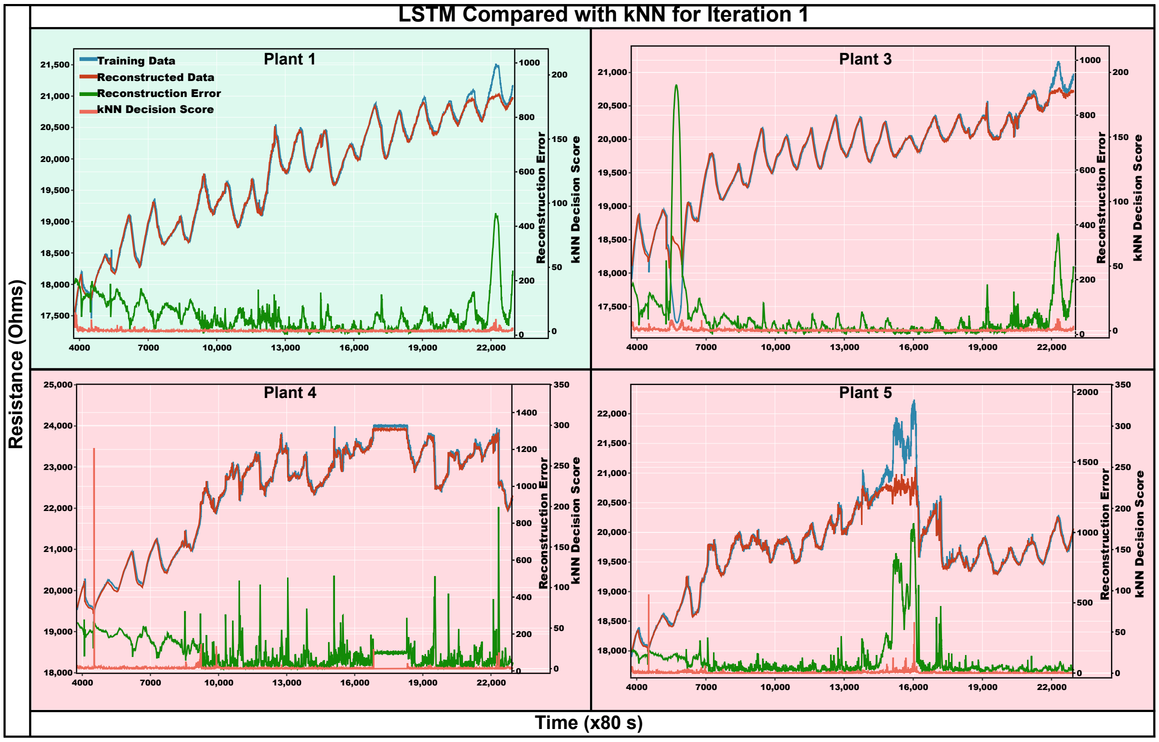

Figure S5. This figure shows the original electrical resistance, reconstructed electrical resistance, calculated kNN decision score and LSTM Reconstruction error for the plants 1, 3, 4, and 5. The LSTM autoencoder was trained on the scaled electrical resistance data of plant 1 (19185 samples) and then stress was predicted on the resistance data of other plants.

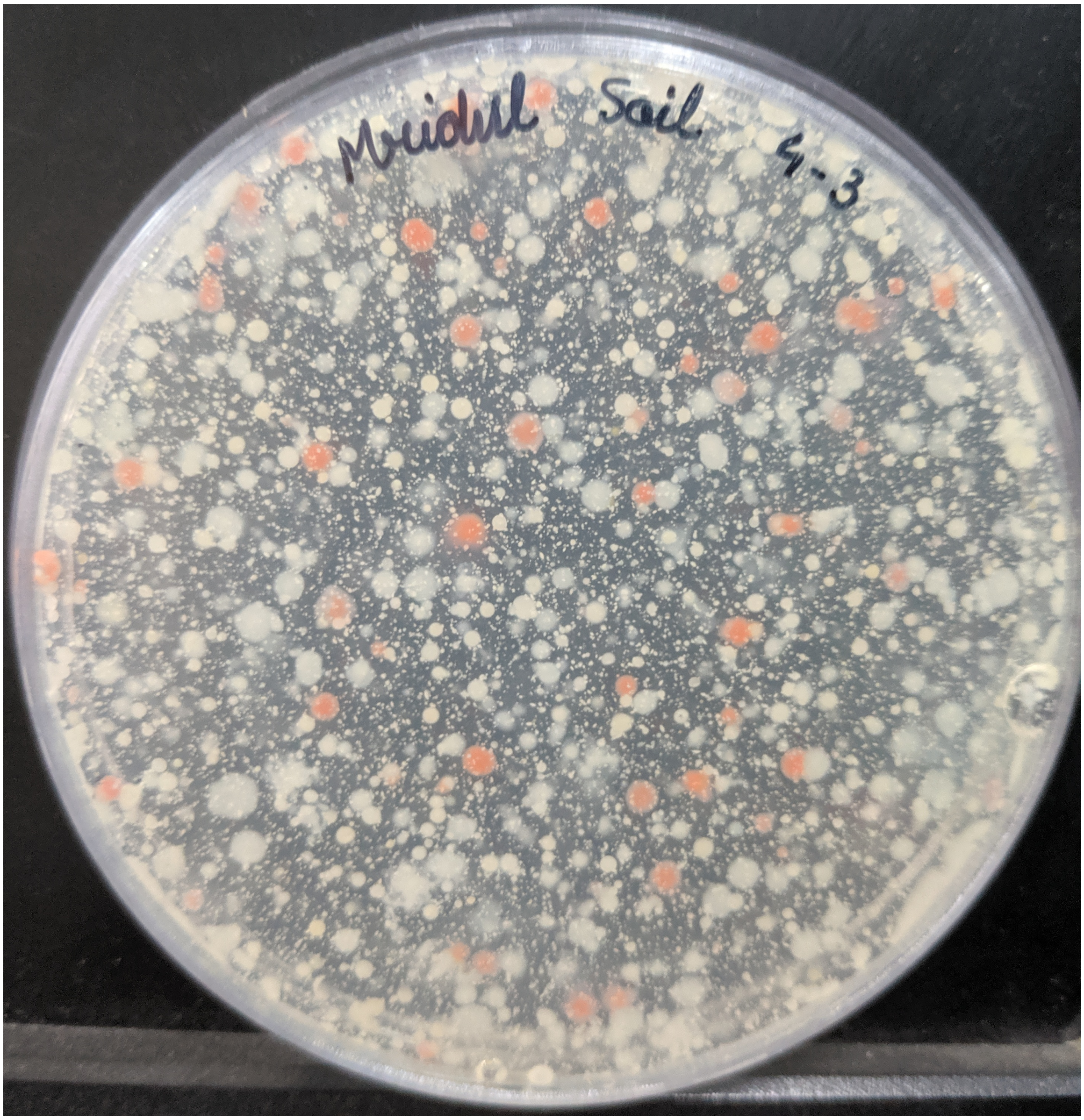

Figure S6. This figure shows the grown colonies on plate count agar from the soil sample which was used to stress the plants in iteration 2 of the experiment. Here, the orange colony can be identified as *Serratia marcesens.*

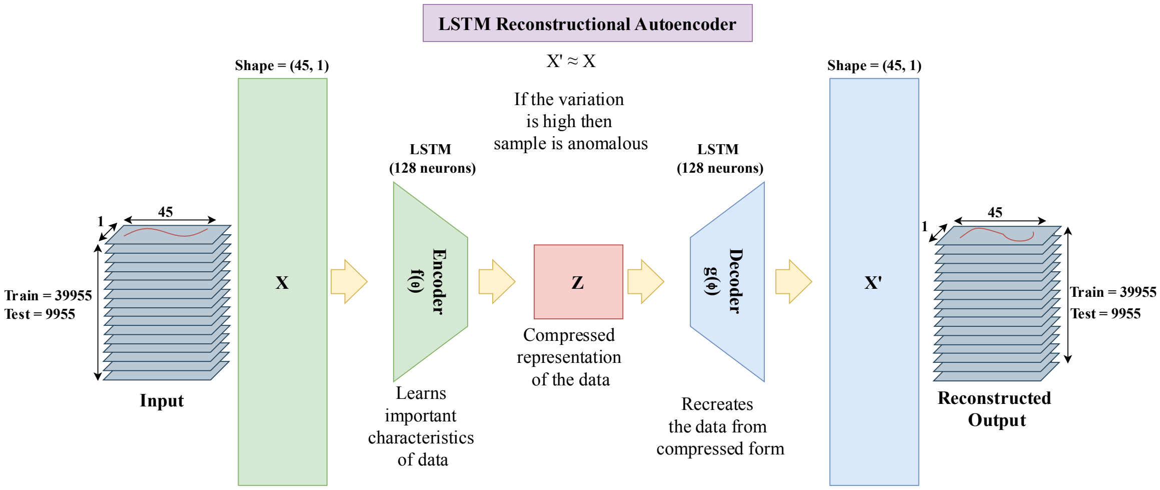

Figure S7. This figure shows the architecture of Long Short-Term Memory (LSTM) autoencoder used for the anomaly detection in the manuscript.

Supplementary Video File

The file (Anomaly Detection.mp4) contains the screen recording of the graphical user interface used to live detect the anomalies in the resistance characteristics of the plants.

Dataset S1 (separate file).

The dataset file contains the experimental data for first and second iteration of the experiment.

SI References

1. Lambert C. Candlestick charts: an introduction to using candlestick charts. Harriman House Limited; 2009.

2. Kim J-S, Chun K-Y, Han C-S. Ion channel-based flexible temperature sensor with humidity insensitivity. Sensors and Actuators A: Physical. 2018;271:139–45.

3. Zhao Y, Nasrullah Z, Li Z. PyOD: A Python Toolbox for Scalable Outlier Detection. Journal of Machine Learning Research. 2019;20(96):1–7.
