# Supplementary material for: Decoding the physiological response of plants to stress using deep learning for forecasting crop loss due to abiotic, biotic, and climatic variables
Source: Sci Rep. 2023 May 26;13:8598. doi: 10.1038/s41598-023-35285-3 (PMC10215062; doi:10.1038/s41598-023-35285-3)
Supplement: Supplementary file 2 — Supplementary Information 2. [file 41598_2023_35285_MOESM2_ESM.pdf]

Decoding the Physiological Response of Plants to Stress using Deep Learning for Forecasting Crop Loss due to Abiotic, Biotic, and Climatic Variables

Scientific Reports

Mridul Kumar[1], Zeeshan Saifi[1] and Krishnananda Soami Daya[1]
[1] Microwave Physics Lab, Department of Physics and Computer Science, Dayalbagh Educational Institute, Dayalbagh, Agra, 282005, Uttar Pradesh, India
Corresponding Author : Prof. Krishnananda Soami Daya (ksdaya@dei.ac.in)

Appendix A

Derivation for relative change in charge carrier concentration

Drude model which fundamentally explains the variation in conductivity of charge carriers detached from the native atom was used for creating a theoretical model to establish a relationship between electrical resistance and plant stress which was measured on the basis of relative change in the nutrient concentration of the growth media. According to Drude model, for electrodes separated by a distance l with cross-section area A the electrical resistance (R_0) of the two electrode system containing electrolytic solution is given by,

$$R_0 = \frac{ml}{nq^2 A\tau} \tag{A1}$$

with τ as the mean relaxation time for n charge carriers in the electrolyte. Rewriting temperature dependence of resistivity (ρ) (Kim, Chun, & Han, 2018) of ionic solution having temperature compensation factor (α) in terms of resistance,

$$R = \frac{R_0}{1 + \alpha(T - T_0)} \tag{A2}$$

Using the value of R_0 from equation (A1) and since m, A, l, q are constant and τ only depends on temperature which did not vary much, so can also be considered constant,

$$R \propto \frac{1}{n[1 + \alpha(T - T_0)]} \tag{A3}$$

So, it can be inferred from equation (A3) that the variation in resistance is caused by change in either the temperature (T) or the number of charge carriers (n).

From our calculations, the estimated value of $\alpha = 0.0141/°\text{C}$ (See Supplementary File Text, Table S1 and S2 for calculations) was applied to the equation (A3).Taking k as the proportionality constant, n can then be given as,

$$n = \frac{k}{R[1 + 0.0141(T - T_0)]} \tag{A4}$$

From equation (A4), the goal is to calculate the relative variation in the charge carrier concentration in the growth media when the plant was stressed compared with when not stressed. Taking the electrical resistance curve of the plant in bottle 2 (any of the bottles can be chosen) for the second iteration (see Fig. ??) of the experiment and plotting it with the corresponding temperature, choosing an arbitrary reference point on the time axis when the plant was not stressed and applying the corresponding temperature (R_0 Ω and T_0 °C) as the reference in equation (A4) we get,

$$n = \frac{k}{R[1 + 0.0141(T - T_0)]} \tag{A5}$$

Equation (A5) can give the amount of charge carrier at any other point on the time axis relative to the reference point. The corresponding resistance is R and the temperature is T (R, T) in terms of k. So, if these values are applied, viz., $T = T_0^{\circ}$C and $R = R_0\Omega$ in equation (A5), we get the charge carrier concentration (n_0) in terms of k at point (R_0 Ω, T_0 °C),

$$n_0 = \frac{k}{R_0} \tag{A6}$$

At any other point on the time axis where the resistance is R,the temperature is T and the charge concentration is n, then equation (A5) would give the charge carriers as,

$$n = \frac{k}{R[1 + 0.0141(T - T_0)]} \tag{A7}$$

Relative change in charge carrier concentration compared to point (19200 Ω and 23.54 °C) can be given as,

$$\frac{n - n_0}{n_0} = \frac{R_0}{R[1 + 0.0141(T - T_0)]} - 1 \tag{A8}$$

Equation (A8) gives the relative change in charge carrier concentration of other points on the time axis with respect to (R_0 Ω, T_0 °C).

References

Kim, J.-S., Chun, K.-Y., Han, C.-S. (2018). Ion channel-based flexible temperature sensor with humidity insensitivity. *Sensors and Actuators A: Physical*, *271*, 139–145. (Publisher: Elsevier)
